# Immunotherapy with STING and TLR9 agonists promotes synergistic therapeutic efficacy with suppressed cancer-associated fibroblasts in colon carcinoma

**DOI:** 10.3389/fimmu.2023.1258691

**Published:** 2023-10-13

**Authors:** Sare Hajiabadi, Soodeh Alidadi, Zohreh Montakhab Farahi, Mohammad M. Ghahramani Seno, Hamidreza Farzin, Alireza Haghparast

**Affiliations:** ^1^ Department of Pathobiology, Faculty of Veterinary Medicine, Ferdowsi University of Mashhad, Mashhad, Iran; ^2^ Program for Genetics and Genome Biology, The Hospital for Sick Children, Toronto, ON, Canada; ^3^ Razi Vaccine and Serum Research Institute, Agriculture Research, Education and Extension Organization (AREEO), Mashhad, Iran

**Keywords:** STING, TLR9, immunoadjuvants, CAFs, colon carcinoma

## Abstract

The innate immune sensing of nucleic acids using effective immunoadjuvants is critical for increasing protective immune responses against cancer. Stimulators of interferon genes (STING) and toll-like receptor 9 (TLR9) agonists are considered promising candidates in several preclinical tumor models with the potential to be used in clinical settings. However, the effects of such treatment on tumor stroma are currently unknown. In this study, we investigated the immunotherapeutic effects of ADU-S100 as a STING agonist and CpG ODN1826 as a TLR9 agonist in a preclinical model of colon carcinoma. Tumor-bearing mice were treated intratumorally on days 10 and 16 post-tumor inoculation with ADU-S100 and CpG ODN1826. Cytokine profiles in the tumor and spleen, tumor cell apoptosis, the infiltration of immune cells, and cancer-associated fibroblasts (CAFs) in the tumor microenvironment (TME) were evaluated to identify the immunological mechanisms after treatment. The powerful antitumor activity of single and combination treatments, the upregulation of the expression of pro-inflammatory cytokines in the tumor and spleen, and the recruitment and infiltration of the TME by immune cells revealed the synergism of immunoadjuvants in the eradication of the colon carcinoma model. Remarkably, the significant downregulation of CAFs in the TME indicated that suppression of tumorigenesis occurred after immunoadjuvant therapy. The results illustrate the potential of targeting the STING and TLR9 pathways as powerful immunoadjuvants in the treatment of preclinical colon carcinoma and the possibility of harnessing these pathways in future therapeutic approaches.

## 1 Introduction

Despite extensive research and recent improvements in prognosis, prevention, and treatment, cancer is a leading cause of death worldwide. Colon carcinoma is the third most prevalent cancer and the second most lethal cancer in the world (1–3).

It is well known that immunotherapy, as a promising strategy, modulates the immune system for the treatment of cancer by stimulating the body’s immune response against tumor growth and metastasis (4). However, there is still a need for the development and improvement of more effective therapies.

In immunotherapy, multiple approaches and different compounds have been studied so far, several of which work by inducing innate immune responses (5, 6). Nucleic acid-based immunoadjuvants are regarded as safe and powerful immunostimulatory compounds in therapeutic and vaccine settings. CpG oligodeoxynucleotides (CpG ODNs) are synthetic ligands of toll-like receptor 9 (TLR9), which is located in the endosomal compartment of cells (7). CpG ODNs, as an adjuvant approved by the U.S. Food and Drug Administration (FDA), demonstrate remarkable efficacy in phase I clinical trials of infectious diseases and cancer (8–10). As a result of CpG ODN binding to the TLR9 receptor, the myeloid differentiation primary response 88–interferon regulatory factor 7 (MyD88–IRF7) pathway is activated and stimulates the production of type I interferons (IFNs), thereby promoting the pro-inflammatory status of cells in a nuclear factor kappa B (NF-κB)-dependent manner (11). Moreover, CpG ODNs can trigger Th1-type immune responses through the polarization of plasmacytoid dendritic cells (pDCs) to produce type I IFNs, thereby enhancing the recruitment of T cells, induction of B cells, and pDC activation, which consequently promotes effector antitumor immunity (8, 12). A class B CpG ODN that is distinct for mouse TLR9 is CpG ODN1826, containing a full phosphorothioate backbone that is nuclease-resistant and has been well studied in several preclinical studies so far (8, 13).

Another well-known immunostimulatory compound is the stimulator of interferon genes (STING), which is present in the endoplasmic reticulum and activated by double-stranded DNA (dsDNA) (14–16). The production of type I interferon and pro-inflammatory cytokines by a variety of cells in the TANK-binding kinase 1–interferon regulatory factor 3 (TBK1–IRF3)- and TBK1– NFκB-dependent pathways is a consequent cascade of STING activation (17, 18), leading to the polarization of antigen-presenting cells (APCs) and the recruitment of cytotoxic T cells (19, 20).

Studies have revealed that DNA sensing by STING has a significant function in the immune recognition of tumors, which is crucial to effective cancer immunotherapy (21).

The application of the STING agonist cyclic GMP-AMP (cGAMP) as an immunoadjuvant in cancer is partially limited because it does not penetrate the cell membrane (22). To overcome the limitations of natural cGAMP, rational modifications of cyclic dinucleotides (CDNs) led to the introduction of a newly synthesized CDN called ML RRS2 CDA (MIW815 or ADU-S100), which had improved features such as high stability and lipophilicity and demonstrated stronger antitumor effects in various cancer models (23, 24). It induced effective tumor regression via intratumoral injection in the B16 melanoma, CT-26 colon cancer, and 4T1 breast cancer models (20, 25–30). In addition to mouse STING, this compound is able to activate all known human STING allelic variants. This agonist shows significant potential as a therapeutic agent to promote tumor microenvironment (TME) activation in many tumor types by activating efficient and lasting antitumor CD8^+^ T cell responses (20).

The TME includes an extracellular matrix and a variety of cells consisting of infiltrating inflammatory cells, endothelial cells, and cancer-associated fibroblasts (CAFs) (31). CAFs form the dominant component of many tumors and promote tumorigenesis and cancer metastasis in several ways, including the production of growth factors, cytokines, and chemokines; the degradation of extracellular matrix (ECM) proteins; the promotion of angiogenesis; and the immunosuppression and prevention of drug penetration (31–35). Recent studies have demonstrated the presence of a heterogeneous population of CAFs in the TME, including tumor-promoting CAFs and tumor-restraining CAFs (34, 35). Different biomarkers such as vimentin (VIM), alpha-smooth muscle actin (α-SMA), fibroblast activation protein (FAP), fibroblast-specific protein 1 (FSP1), and platelet-derived growth factor receptor (PDGFR), have been used to identify CAFs (33, 35).

Myofibroblasts’ contractile abilities, led by the expression of α-SMA, which is an isoform of actin, induce a positive feedback loop that amplifies the fibrotic cycle, which in turn results in tumor growth (36). There are different methods for detecting CAFs and their heterogenicity in tissues, which corresponds to their biological functions and subtypes. The CAFs are clustered into three subpopulations, namely antigen-presenting CAFs (apCAFs), myofibroblastic CAFs (myCAFs), and inflammatory CAFs (iCAFs) (37–39). Single-cell RNA-sequencing, multiplex immunostaining, and genetic mouse models have been used for the identification and biological functions of different subtypes of CAFs in pancreatic ductal adenocarcinoma (PDAC), colorectal cancer (CRC), breast cancer, and liver fibrosis progression (40–44).

Although several recent studies have highlighted the importance of combination therapy using STING and TLR agonists in preclinical cancer models (45–48), the relationship between such immunoadjuvants and CAFs as key players in stromal tumorigenesis has not yet been determined. Therefore, in this study, the synergistic effects of STING and TLR9 agonists and the immunological mechanisms underlying tumor regression and CAFs’ infiltration of the TME were studied in a preclinical colorectal tumor model. In addition, several important cellular markers of immunity, pro-inflammatory cytokine expression, apoptosis, and pro-tumorigenesis were studied.

Our results suggest that the combination of ADU-S100 and CpG ODN1826 shows synergistic therapeutic effects with enhanced antitumor immune responses and suppressed tumorigenesis markers and, therefore, can be considered a promising target for further cancer immunotherapy investigation.

## 2 Materials and methods

### 2.1 Cell line

The CT-26 cell line was purchased from The Research Institute of Biotechnology (Mashhad, Iran) and cultured in Roswell Park Memorial Institute (RPMI) 1640 medium (Gibco^®^, Grand Island, NE, USA), supplemented with 10% heat-inactivated fetal bovine serum (Gibco), 2 mM L-glutamine (Invitrogen, San Diego, USA), 25 mM β-mercaptoethanol (Merck, Munich, Germany), and 100 IU/mL penicillin/streptomycin (Gibco) at 37°C and in a 5% CO_2_ atmosphere.

### 2.2 Animals

Female BALB/c mice (6–8 weeks old) were obtained from the Royan Institute (Tehran, Iran). All the animals were kept at the animal housing of the Faculty of Veterinary Medicine, Ferdowsi University of Mashhad, Mashhad, Iran. The ***in vivo*** experiments were reviewed, approved, and conducted in accordance with the ethics guidelines of the Animal Care and Use Committee of Ferdowsi University of Mashhad (license number 46013-M).

### 2.3 *In vivo* tumor model

The CT-26 adenocarcinoma cells (3 × 10^5^) were injected subcutaneously (SC) into the right flank of the mouse in 100 µL of phosphate-buffered saline (PBS). Every other day, the tumor size was measured with a digital caliper for a total of 30 days, and the tumor volume was calculated using the equation 1:

V = (L × W^2^)/2 (1)

where the letter “L” represents the large diameter and “W” represents the small diameter of the tumor (49, 50). The mice were divided into five groups (*n* = 7), as depicted in **Table 1**, and treatment with ADU-S100 (MedChemExpress, South Brunswick Township, NJ, USA) and CpG ODN1826 (InvivoGen, San Diego, CA, USA) was administered intratumorally on days 10 and 16 post tumor inoculation. The mice were euthanized on day 30 post-tumor induction and their body weights were recorded. Subsequently, the tumors were extracted and weighed. After conducting a necropsy of the mice, samples of their tumor, spleen, and liver tissues were taken. The tumor inhibition rate was calculated using the equation 2 (6):

**Table 1:** Treatment groups used in this study.

Treatment	Dose per mouse
**PBS**	**–**
**ADU-S100**	**20 µg**
**ADU-S100**	**40 µg**
**CpG ODN1826**	**40 µg**
**ADU-S100 + CpG ODN1826**	**20 µg + 20 µg**

PBS, phosphate-buffered saline.

Tumor inhibition rate (%) = [(mean tumor weight of control group − mean tumor weight in treated group)/mean tumor weight of control group] × 100% (2).

The spleen index was calculated using the equation 3 (51):

Spleen index = weight of spleen (mg)/body weight (g). (3)

### 2.4 RNA extraction and quantitative real-time polymerase chain reaction analysis

Total RNA was extracted from the spleen and tumor tissues using a total RNA isolation kit (DENAzist Asia, Mashhad, Iran). The extracted total RNA was subjected to 1U of DNase I (RNase free) (Thermo Scientific Fisher, Waltham, MA, USA) at 37°C for 15 **min**. The inactivation of the DNase was conducted by adding 1 μL of 50 mM ethylenediaminetetraacetic acid (EDTA) (Merck). Moloney murine leukemia virus (M-MLV) reverse transcriptase (Thermo Fisher Scientific) was used to convert RNA samples to cDNA. The quantitative real-time polymerase chain reactions (qRT-PCR) were carried out using RealQ Plus 2x Master Mix Green (Ampliqon A/S, Odense, Denmark) in a Rotor-Gene^®^ Q real-time PCR cycler (QIAGEN, Hilden, Germany). The amplification steps were as follows: 15 **min** at 95°C (1 cycle), followed by 30 s at 95°C, 30 s at 58°C–62°C, and 25 s at 72°C (35–40 cycles). Three replicates were considered for qRT-PCR readout. The β-actin gene served as the internal control. **Table 2** gene-specific primers and their accession numbers used in this study.

**Table 2:** Gene-specific primer sequences used in the qRT-PCR analysis.

Gene	Primer sequence	Product (bp)	Annealing temperature (°C)	Accession number
β-actin	5´–TTCGCCATGGATGACGATATC–3´ 3´–GGCCTCGTCACCCACATAG–5´	180	58 60	NM_007393.5
IFN-β	5´–GATGAACTCCACCAGCAGACA–3´ 3´–CACCATCCAGGCGTAGCTG–5´	168	60 60	NM_010510.1
IFN-γ	5´–CTGCGGCCTAGCTCTGAGAC–3´ 3´–CTGGCTCTGCAGGATTTTCATG–5´	228	62 60	NM_008337.4
TNF-α	5´–GCCACCACGCTCTTCTGTCTA–3´ 3´–GAGGGTCTGGGCCATAGAAC–5´	105	62 60	NM_001278601.1
IL-12	5´–GTTGGAAGCACGGCAGCAG–3´ 3´–AGGGAGAAGTAGGAATGGGGAG–5´	177	62 60	NM_001303244.1
IL-6	5´–GGATACCACTCCCAACAGACC–3´ 3´–GTTTTCTGCAAGTGCATCATCG–5´	148	60 59	NM_001314054.1

IFN-β, interferon beta; IFN-γ, interferon-gamma; TNF-α, tumor necrosis factor alpha; IL-12, interleukin 12; IL-6, interleukin 6; qRT-PCR, quantitative real-time polymerase chain reaction.

### 2.5 Hematoxylin and eosin staining

The samples obtained from the tumor, liver, and spleen tissues were fixed in 10% neutral buffered formalin (Merck) for 24 **h**. The 4-μm-thick sections of the tissue samples were stained with hematoxylin and eosin (H&E) (Merck), mounted on the slide, and finally, examined under a light microscope (Labomed Inc., Los Angeles, CA, USA). The mitotic cells were counted in 10 fields of the tumor slides, and, subsequently, the mean number of mitotic cells was calculated.

### 2.6 Immunohistochemical staining of tumor tissues

Sections of formalin-fixed paraffin-embedded tumor tissues were prepared. The sections were deparaffinized and rehydrated, and the retrieval of antigens was carried out using Tris-ethylenediaminetetraacetic acid (EDTA) buffer (10 mM Tris base, 1 mM EDTA solution, 0.05% Tween 20, pH 9.0) (Merck) for 20 min at 98°C. Subsequently, the slides were quenched using 0.3% [volume to volume (v/v)] methanol-diluted hydrogen peroxide (Merck). The sections were incubated for 30 min with different concentrations of primary antibodies, e.g., anti-CD8-α (1 out of 200) (cat. number sc-7970; RRID: AB_627208; Santa Cruz Biotechnology, Dallas, TX, USA), anti-cleaved caspase 3 (1 out of 300) (cat. number 9661; RRID: AB_2341188; Cell Signaling Technology, Danvers, MA, USA), anti-vimentin (1 out of 400) (cat. number 5741; RRID: AB_10695459; Cell Signaling Technology), anti-CD45 (1 out of 3,200) (cat. number sc-53665, RRID: AB_629093; Santa Cruz Biotechnology), and anti-α-SMA (1 out of 500) (cat. number 65001, RRID: AB_2920672; Progen, Heidelberg, Germany). After washing with Tris-buffered saline (TBS) (0.5M Tris base, 9% NaCl, pH 8.4) (Merck), the slides were incubated with secondary antibodies in accordance with the protocol. Images of the stained slides were obtained using a light microscope from 10 different fields of high-power fields (×400 overall magnification) and then analyzed using QuPath software (RRID: SCR_018257). The caspase-3 labeling index was calculated using the following equation (52):

Caspase-3 labeling index = (number of activated caspase-3-positive cells × 100)/total number of nuclei. (4)

### 2.7 Measurements of hematological parameters

Before the animals were euthanized on day 30, whole blood was immediately collected from the hearts of the mice in a tube containing an anticoagulant. A complete blood count (CBC) analysis was performed using a cell counter (Nihon Kohden, Nima Pouyesh Teb, Iran).

### 2.8 MTT test

The CT26 cells were seeded in 96-well culture plates at 7 × 10^3^ cells per well. The cells were incubated with different concentrations of ADU-S100, CpG ODN (10 μg/mL, 20 μg/mL, 40 μg/mL, and 60 μg/mL), or ADU-S100 (20 μg/mL) + CpG ODN (20 μg/mL) at 37°C for 24 h. The cells were further incubated with 10 μL per well of 3-(4,5-dimethylthiazol-2-yl)-2,5-diphenyl-2H-tetrazolium bromide (MTT) (5 mg/mL; Sigma, USA) for 3 h. Subsequently, the medium was removed and 100 μL per well of dimethyl sulfoxide (DMSO) was added. The absorbance was measured at 490 nm. The experiment was conducted in triplicate (*n* = 3).

### 2.9 Statistical analysis

Statistical analysis was conducted using GraphPad Prism software version 9.0 (RRID: SCR_002798; GraphPad Software Inc., San Diego, CA, USA). One-way analysis of variance and Mann–Whitney U–tests were used to analyze the data. The results are shown as mean ± SD and a *p*-value ≤ 0.05 is considered to be statistically significant. The relative expression software tool version 2.0.13 (REST 2009; RRID: SCR_023755) was used to analyze the qRT-PCR results by way of a pairwise fixed reallocation randomization test. Significant differences among the groups were indicated as ****p ≤ 0.0001, ***p ≤ 0.001, **p ≤ 0.01, and *p ≤ 0.05.

## 3 Results

### 3.1 Inhibition of tumor growth and increased survival rate of colon adenocarcinoma-bearing mice by single or combination therapy with CpG ODN1826 and ADU-S100

**Figure 1:**
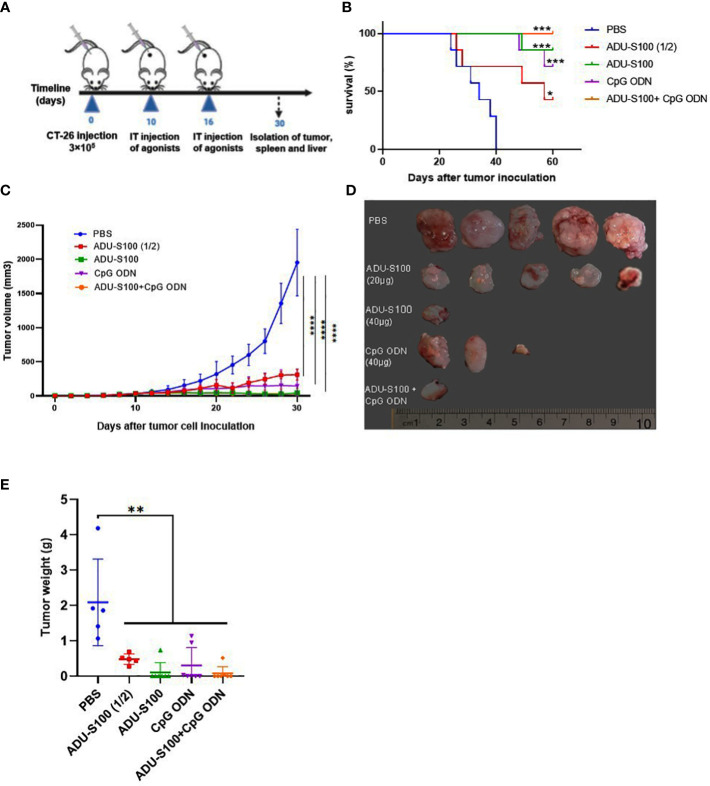
Intratumoral (IT) administration of ADU-S100 and CpG ODN1826 reduces tumor volume in the CT-26 adenocarcinoma model. **(A)** Mice were injected subcutaneously with 3 × 10^5^ CT-26 cells (in 100 μL of PBS) on day 0. On days 10 and 16, mice were given IT injections of PBS, ADU-S100 (20 µg or 40 µg), CpG ODN (40 µg), ADU-S100 (20 µg) + CpG ODN (20 µg) (*n* = 7). **(B)** Kaplan–Meier survival curve of the animals. **(C)** Tumor volumes were monitored every other day for 30 days. **(D)** Photographs of dissected tumor tissues from treated mice on day 30 of treatment. **(E)** Average tumor weight in different treatment groups at the end of the experiment. Data are presented as mean ± SD. Significant differences in the survival plot were measured using a weighted log-rank test. Other data were analyzed using one-way ANOVA and Mann–Whitney tests. Statistically significant differences are indicated as ****p ≤ 0.0001, ***p ≤ 0.001, **p ≤ 0.01, and *p ≤ 0.05. PBS, phosphate-buffered saline.

ADU-S100 is a STING agonist with a potent antitumor effect that has been validated in several preclinical tumor models. We hypothesized that the combination of ADU-S100 and CpG ODN 1826 at a reduced (i.e., 50% lower) concentration, compared with a single form of the agonist, would have an additive and robust anticancer effect in suppressing the tumor growth in a CT-26 colon cancer model. The cytotoxic effects of a single, or a combination of, agonist(s) were evaluated using an MTT assay. ADU-S100 and CpG ODN1826, alone or in combination, did not show any adverse effects on the viability of CT-26 cells *in vitro* (**Figure S1**). The injection of a CT-26 cell suspension was administered subcutaneously into the right flanks of the mice. When palpable tumors appeared, mice were treated with intratumoral injections of PBS, ADU-S100 (20 μg and 40 μg), CpG ODN1826 (40 μg), or a combination of ADU-S100 (20 μg) and CpG ODN1826 (20 μg). Every other day, tumor volumes and mouse weights were recorded. To evaluate the immunological mechanisms underlying tumor regression, in a second experiment, all mice were euthanized on day 30 post tumor inoculation (**Figure 1A**). The standard survival curve (Kaplan–Meier), which describes the direct effects of treatment on tumor growth, showed that both single and combination therapy were able to prolong the survival rate of tumor-bearing mice. Notably, the combination treatment eradicated all CT-26 tumors. Specifically, the combination treatment group exhibited a higher survival rate (100%) than the single treatment groups treated with ADU-S100 (40 μg) (86%), CpG ODN (40 μg) (71%), and ADU-S100 (20 μg) (43%) (**Figure 1B**). **Figures 1C, D** illustrate how the tumor volume in all treatment groups was significantly reduced in comparison to the control group. The combination group [ADU-S100 (20 μg) + CpG ODN (20 μg)] showed the highest growth inhibition, from 1,952 mm^3^ to 32 mm^3^, and was therefore more effective than a single injection of each compound. In addition, in the ADU-S100 (40 μg) group, the tumor regression size reached 44.8 mm^3^ and 144 mm^3^ in the CpG ODN (40 μg) treatment group. These results indicate that the antitumor effects of ADU-S100 when combined with CpG ODN are approximately equal to those of monotherapy with ADU-S100 when administered at higher doses (i.e., at twice the concentration). The average tumor weight was significantly reduced from 2.09 g in the control group to 0.08 g and 0.1 g in the combination and the ADU-S100 (40 μg) groups, respectively (**Figure 1E**). The curve of the tumor inhibition rate is displayed in **Figure S2A**. The results show that ADU-S100 exhibited powerful antitumor activity that is dose-dependent, meaning that ADU-S100 at 20 µg suppressed CT-26 tumor growth by 77% on day 30, whereas for the 40-µg dose, the tumor inhibition rate was 95%. Furthermore, the combination of ADU-S100 and CpG ODN1826 showed the highest tumor inhibition rate (96%), whereas in the CpG ODN (40-µg) group, the tumor inhibitory rate was decreased to 85%. In the second *in vivo* experiment, on day 30 post-tumor inoculation, mice were sacrificed, and their spleens and tumors were removed and weighed for further immunological analysis. Owing to the importance of the spleen as an extramedullary hematopoietic tissue that produces immunosuppressive myeloid cells in tumor-bearing mice (53), we measured the spleen index (mg/g) in this study. The curve of the spleen index is presented in **Figure S2B** and shows that the spleen index was positively correlated with tumor weight. Furthermore, during the immunotherapy period, the body weight of the mice was recorded, and no considerable weight change was observed compared with the control group (**Figure S2C**). Overall, these data suggest that either the ADU-S100 treatment or the combination treatment had a profound inhibitory effect on the tumor growth progression, but that the combination of ADU-S100 (20 μg) + CpG ODN (20 μg) at a reduced concentration (i.e., 50% lower) was able to effectively control tumor growth and significantly prolong the survival of CT-26 tumor-bearing mice.

### 3.2 Upregulation of pro-inflammatory cytokine expression in tumor tissues

**Figure 2:**
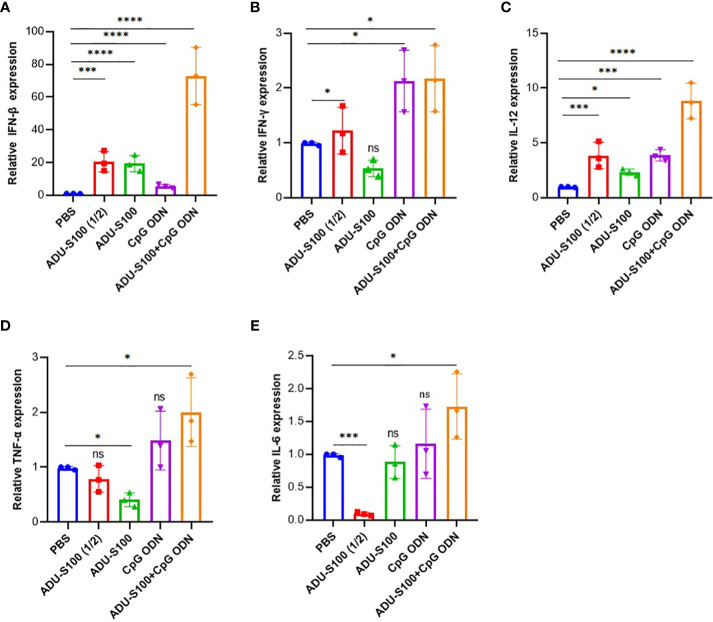
Expression profile of pro-inflammatory cytokines in the tumor. Quantitative real-time polymerase chain reaction (qRT-PCR) analysis of **(A)** IFN-β, **(B)** IFN-γ, **(C)** TNF-α, **(D)** IL-12, and **(E)** IL-6 in tumor tissues of treated mice on day 30 post tumor inoculation (*n* = 3 independent experiments). The transcripts for each gene were quantified relative to β-actin expression levels in each group. Data are presented as mean ± SD. Data were analyzed using REST software and statistically significant differences are indicated as ****p ≤ 0.0001, ***p ≤ 0.001, and *p ≤ 0.05. IFN-β, interferon beta; IFN-γ, interferon-gamma; IL-12, interleukin 12; IL-6, interleukin 6; qRT-PCR, quantitative real-time polymerase chain reaction; TNF-α, tumor necrosis factor*-*alpha.

The induction of pro-inflammatory cytokine expression by CpG ODNs and CDNs has already been reported (46, 54). To determine whether ADU-S100 and CpG ODN1826 could induce effective immune responses and generate antitumor immunity, we evaluated the expression of several key pro-inflammatory cytokines (e.g., IFN-β, IFN-γ, TNF-α, IL-12, and IL-6) using qRT-PCR analysis. We observed that in tumor tissues, the combination treatment [ADU-S100 (20 μg) + CpG ODN (20 μg)] upregulated the expression level of IFN-β, IL-12, and TNF-α in a synergistic manner compared with the other treatments (**Figures 2A–E**). All treatment groups displayed a significant upregulation of IFN-β expression compared with the control group (*p* ≤ 0.05) (**Figure 2A**). The expression level of IFN-γ, as one of the most potent cytokine hallmarks of antitumor immunity, was significantly upregulated in the combination [ADU-S100 (20 μg) + CpG ODN (20 μg)] and CpG ODN groups compared with the control group (*p* ≤ 0.05) (**Figure 2B**). In our experiment, IL-12 expression was significantly upregulated in all treatment groups compared with the control group (*p* ≤ 0.05) (**Figure 2C**). In addition to the combination group, which showed the highest level of expression of TNF-α and IL-6 compared with the control group, the CpG ODN group also showed increased expression of these cytokines compared with the control group, although the difference was not significant (**Figures 2D, E**). These results demonstrated that immunoadjuvant therapy with ADU-S100 and CpG ODN promotes the robust and synergistic expression of several important pro-inflammatory and immune-effector cytokines in CT-26 tumor tissues.

### 3.3 Upregulation of pro-inflammatory cytokine expression in the spleen

**Figure 3:**
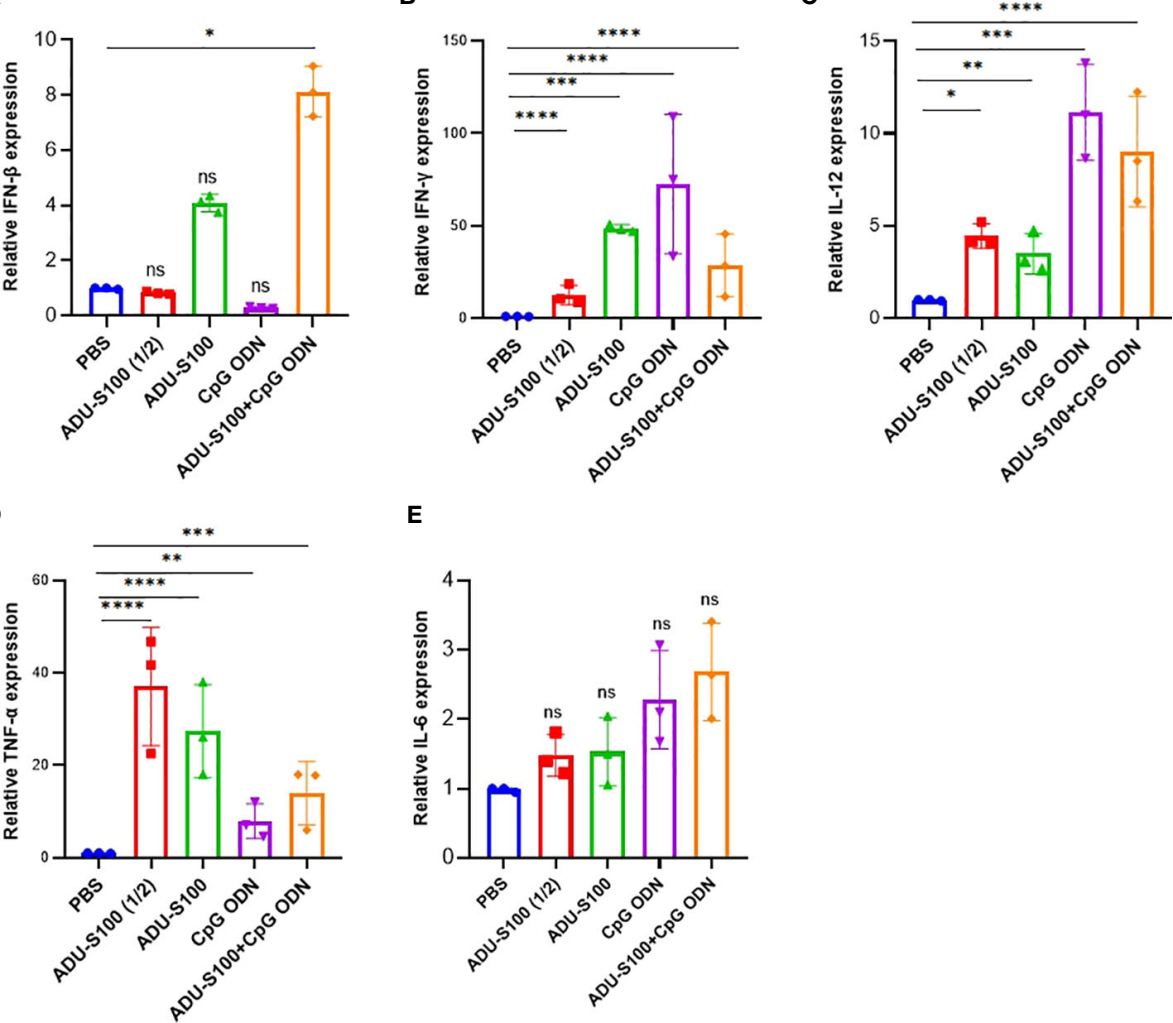
Expression profile of pro-inflammatory cytokines in the spleen. We conducted qRT-PCR analysis of **(A)** IFN-β, **(B)** IFN-γ, **(C)** TNF-α, **(D)** IL-12, and **(E)** IL-6 in the spleen tissues of treated mice on day 30 post-tumor inoculation (*n* = 3 independent experiments). The expression levels were quantified relative to β-actin as a reference gene. Data are presented as mean ± SD. Data were analyzed by REST software and statistically significant differences are indicated as ****p ≤ 0.0001, ***p ≤ 0.001, **p ≤ 0.01, and *p ≤ 0.05. IFN-β, interferon beta; IFN-γ, interferon-gamma; IL-12, interleukin 12; IL-6, interleukin 6; qRT-PCR, quantitative real-time polymerase chain reaction; REST, relative software expression tool; TNF-α, tumor necrosis factor*-*alpha.

The spleen is the largest lymphoid organ in the body and contains many immune cells with a crucial role in immune response. In addition to the tumor, we measured the expression profile of pro-inflammatory cytokines (IFN-γ, IFN-β, TNF-α, IL-12, and IL-6) in the spleen tissue as the main secondary host lymphoid compartment (**Figure 3**). In the spleen, the highest level of expression of IFN-β and IL-6 was observed in the combination group [ADU-S100 (20 μg) + CpG ODN (20 μg)], which was increased compared with the control group (**Figures 3A, E**). The group treated with CpG ODN displayed the highest level of expression of IFN-γ and IL-12 compared with the control group (*p* ≤ 0.05) (**Figures 3B, C**). The highest level of TNF-α expression was observed in the ADU-S100 (20 µg) group, which displayed a larger tumor size than the other treatment groups (**Figure 3D**).

### 3.4 Immunohistochemical and histological analysis of tumor tissues showed typical features of apoptosis and inflammation after immunotherapy

**Figure 4:**
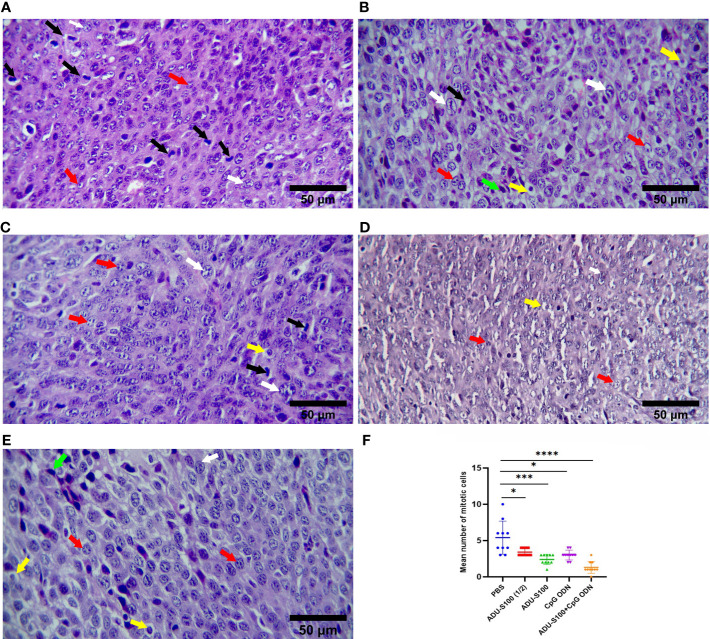
Microphotographs of tumor tissues in the **(A)** control, **(B)** ADU-S100 (20 µg), **(C)** ADU-S100 (40 µg), **(D)** CpG ODN (40 µg), and **(E)** ADU-S100 (20 µg) + CpG ODN (20 µg) groups. Scale bars = 50 μm. (**A–E**, H&E staining, ×400 magnification). Tumor cells undergoing mitosis (black arrows), apoptotic tumor cells (yellow arrows), pleomorphism of the tumor cells (white arrows), tumor cells with one or more nucleoli (red arrows), and mononuclear inflammatory cells infiltrating into the tumor tissue (lymphocytes, green arrows) are shown. **(F)** The mean number of mitotic cells in the tumor tissues (*n* = 3). Statistically significant differences were analyzed using Mann–Whitney tests and indicated as ****p ≤ 0.0001, ***p ≤ 0.001, and *p ≤ 0.05. H&E, hematoxylin, and eosin.

To explore the primary effects of immunoadjuvants in tumor tissue, H&E staining was performed. The tumor cells were characterized by scarce eosinophilic cytoplasm with a round, polygonal, spindle-like polymorphic nucleus of variable size. They contained one or more nuclei, within a scarce stroma (**Figure 4**). The tumor tissues of all mice treated with ADU-S100 (20 µg and 40 µg), CpG ODN (40 µg), and combination groups [ADU-S100 (20 μg) + CpG ODN (20 μg)], showed typical features of apoptosis and inflammation, indicating the effectiveness of the treatment in tumor clearance (**Figures 4B–E**). The number of mitotic cells in the control group was significantly increased compared with those in the treatment groups (**Figure 4F**). In addition, features of malignancy, such as hemorrhage and necrosis, were observed in the control group (**Figure S3A**). However, no tumor malignancies were observed in the treatment groups (**Figures S3B–E**).

**Figure 5:**
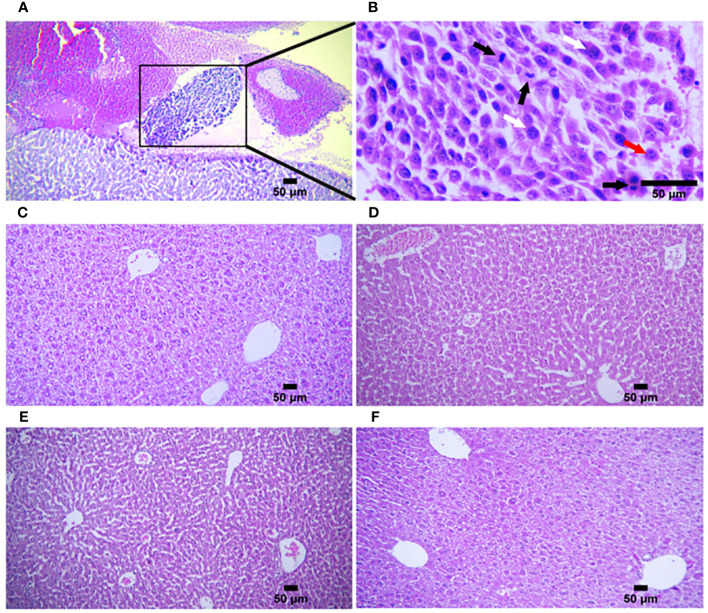
Histologic structures of liver tissues in the control and treatment groups. **(A)** Liver metastasis was observed in only one mouse in the control group (*n* = 5) (H&E staining, ×100 magnification). **(B)** Higher magnification of **(A)** In the metastatic tumor tissue, pleomorphic tumor cells (white arrows) have a vesicular nucleus containing one or more nucleoli (red arrow) with several mitotic figures (black arrows) (H&E staining, ×400 magnification). Liver tissues from **(C)** ADU-S100 (20 µg), **(D)** ADU-S100 (40 µg), **(E)** CpG ODN (40 µg), and **(F)** ADU-S100 (20 µg) + CpG ODN (20 µg) groups show normal structure without metastasis (*n* = 5 for ADU-S100 (20 µg) and *n* = 7 for other treatment groups). Scale bars = 50 μm. (C–F, H&E staining, ×100 magnification). H&E, hematoxylin, and eosin.

**Figure 6:**
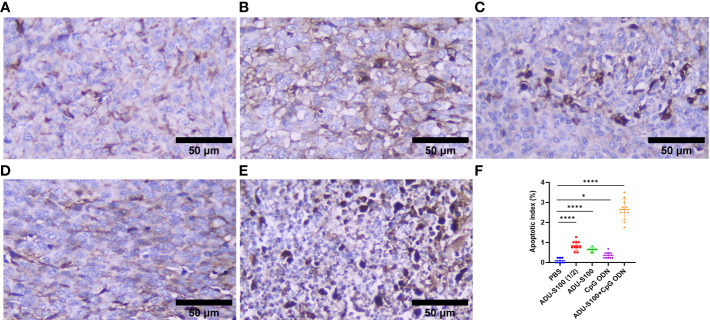
Cleaved caspase-3 expression obtained using IHC in tumor tissues. **(A)** PBS, **(B)** ADU-S100 (20 µg), **(C)** ADU-S100 (40 µg), **(D)** CpG ODN (40 µg), **(E)** ADU-S100 (20 µg) + CpG ODN (20 µg), and **(F)** quantitative analysis of the apoptotic index (*n* = 3). Data are presented as mean ± SD. Statistically significant differences were analyzed using Mann–Whitney tests and indicated as ****p ≤ 0.0001 and *p ≤ 0.05. Scale bars = 50 μm. IHC, immunohistochemistry; PBS, phosphate-buffered saline.

Histopathological analysis of the liver and spleen was performed using H&E staining to assess the probability of tumor metastasis and toxicity linked to the treatment. The microstructure of the spleen in the control and all treatment groups was normal with no histological changes. The liver tissue displayed metastasis in control mice (**Figures 5A, B**), but treatment groups were normal (**Figures 5C–F**). To confirm that the necrotic cells observed in H&E-stained sections were apoptotic, cleaved caspase-3 expression was detected in the tumor tissues using immunohistochemistry (IHC). The results showed a remarkable and highly significant increase of cleaved caspase-3 as the main apoptotic marker in the combination group [ADU-S100 (20 μg) + CpG ODN (20 μg)]. Furthermore, we observed a significant caspase-3 increase in the single-agonist treatment groups compared with the control group (**Figure 6**).

### 3.5 Immunohistochemical analysis showed the abundant infiltration of tumor tissues by CD45^+^ and CD8^+^ cells after immunotherapy

**Figure 7:**
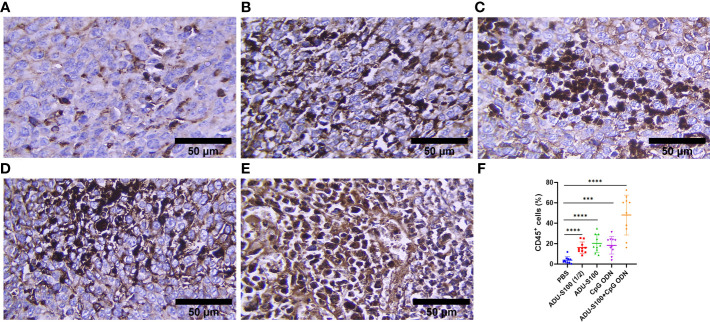
CD45 expression analyzed using IHC in tumor tissues. **(A)** PBS, **(B)** ADU-S100 (20 µg), **(C)** ADU-S100 (40 µg), **(D)** CpG ODN (40 µg), **(E)** ADU-S100 (20 µg) + CpG ODN (20 µg), and **(F)** quantitative analysis of CD45^+^ cells (*n* = 3). Data are presented as mean ± SD. Statistically significant differences were analyzed using Mann–Whitney tests and indicated as ****p ≤ 0.0001 and ***p ≤ 0.001. Scale bars = 50 μm. IHC, immunohistochemistry; PBS, phosphate-buffered saline.

**Figure 8:**
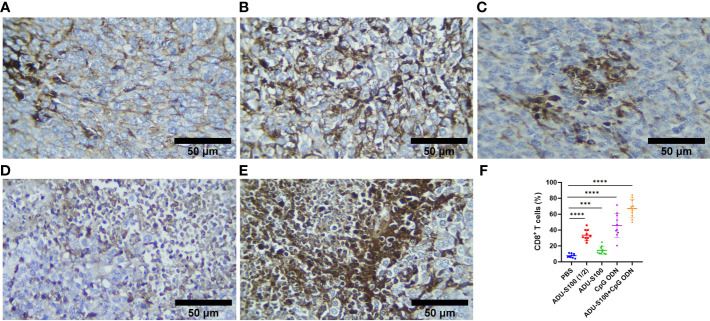
CD8 expression analyzed using IHC in tumor tissues. **(A)** PBS, **(B)** ADU-S100 (20 µg), **(C)** ADU-S100 (40 µg), **(D)** CpG ODN (40 µg), **(E)** ADU-S100 (20 µg) + CpG ODN (20 µg), and **(F)** quantitative analysis of CD8^+^ T cells (*n* = 3). Data are presented as mean ± SD. Statistically significant differences were analyzed using Mann–Whitney tests and indicated as ****p ≤ 0.0001 and ***p ≤ 0.001. Scale bars = 50 μm. IHC, immunohistochemistry; PBS, phosphate-buffered saline.

To assess how ADU-S100 and CpG ODN1826 eradicated tumor growth and increased mice survival rate, immune cell infiltration of the tumor tissues was performed using IHC. CD45, as the most important marker of all nucleated hematopoietic cells (except platelets and erythrocytes), was used to sort the immune cells by IHC. The results showed that in all treatment groups, the level of CD45 expression was significantly increased compared with that of the control group (*p* ≤ 0.05). However, the most abundant expression of this biomarker was observed in the combination group represented (**Figure 7**). Furthermore, due to the crucial role of CD8^+^ T cells in antitumor activity, the expression of this cellular marker was determined using IHC, which exhibited a significant upregulation in the recruitment of CD8^+^ cells in tumor tissues in all treatment groups. The greatest increase in recruitment was observed in the combination group [ADU-S100 (20 μg) + CpG ODN (20 μg], compared with the control group (*p* ≤ 0.05), followed by the CpG ODN (40 μg), ADU-S100 (20 μg), and ADU-S100 (40 μg) groups, respectively (**Figure 8**). These results were consistent with a CBC analysis that showed that the number of lymphocytes in the combination group was significantly increased compared with the control group (*p* ≤ 0.05). In addition, in the CpG ODN (40 µg) and ADU-S100 (20 µg) groups, the level of total blood lymphocytes exhibited a greater than twofold increase compared with the control group (**Figure S4**).

### 3.6 Synergistic effect of ADU-S100 and CpG ODN in the suppression of CAFs’ infiltration of tumor tissues

**Figure 9:**
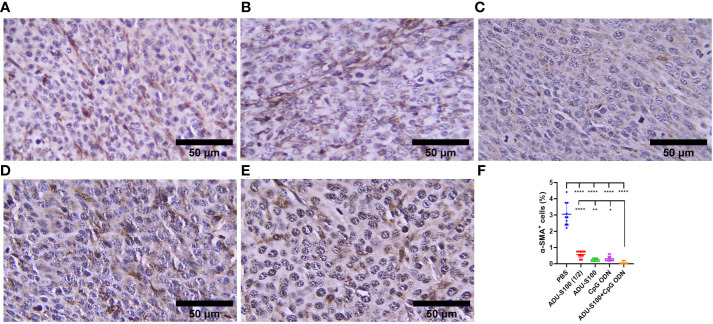
α-SMA expression analyzed by IHC in tumor tissues. **(A)** PBS, **(B)** ADU-S100 (20 µg), **(C)** ADU-S100 (40 µg), **(D)** CpG ODN (40 µg), **(E)** ADU-S100 (20 µg) + CpG ODN (20 µg), and **(F)** quantitative analysis of α-SMA^+^ cells (*n* = 3). Data are presented as mean ± SD. Statistically significant differences were analyzed using Mann–Whitney tests and indicated as ****p ≤ 0.0001, **p ≤ 0.01 and *p ≤ 0.05. Scale bars = 50 μm. IHC, immunohistochemistry; PBS, phosphate-buffered saline.

**Figure 10:**
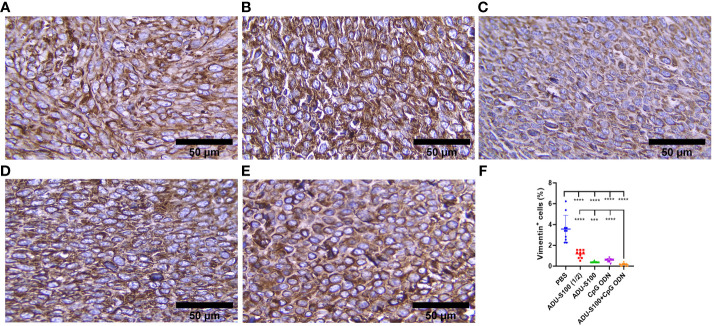
Vimentin expression analyzed by IHC in tumor tissues. **(A)** PBS, **(B)** ADU-S100 (20 µg), **(C)** ADU-S100 (40 µg), **(D)** CpG ODN (40 µg), **(E)** ADU-S100 (20 µg) + CpG ODN (20 µg), and **(F)** quantitative analysis of vimentin^+^ cells (*n* = 3). Data are presented as mean ± SD. Statistically significant differences were analyzed using Mann–Whitney tests and indicated as ****p ≤ 0.0001 and ***p ≤ 0.001. Scale bars = 50 μm. IHC, immunohistochemistry; PBS, phosphate-buffered saline.

CAFs, as the most important stromal cells in the TME, contribute to tumor growth and metastasis through the production of growth factors and cytokines (31). To address the effects of ADU-S100 and CpG ODN on CAF abundance, the expression of α-SMA and VIM were assessed in the formalin-fixed paraffin-embedded tumor tissues using IHC (**Figures 9**, **10**). The results indicated that the expression of these CAF biomarkers was significantly reduced in all treatment groups in comparison to the control group. This reduced expression of α-SMA and VIM was highly significant in the combination group [ADU-S100 (20 μg) + CpG ODN (20 μg)] compared with the other treatment groups. Therefore, the combined effect of the two agonists was remarkably effective in increasing tumor eradication and inhibiting the infiltration of the TME by tumor-supporting cells.

## 4 Discussion

Despite the recent advances and progress made in our understanding of cancer immunotherapy, the field still faces many challenges. Preclinical tumor models, the TME, and their expressed and secreted molecules are considered essential components to better understanding anticancer targeted therapy. This study revealed the immunotherapeutic effects of powerful innate immunity agonists in a mouse model of colon cancer. Tumor-bearing mice were treated intratumorally on days 10 and 16 post-tumor inoculation with ADU-S100, as a STING agonist, and CpG ODN1826, as a TLR9 agonist; both of these are powerful, safe, and effective immunoadjuvants with clinically translational potential. Several mechanistic studies, including on cytokine profiles, immune cells, and CAFs’ infiltration of the TME, were performed to investigate the immunological mechanisms of tumor eradication and the extended survival rate after treatment. The results revealed the powerful antitumor activity of combination treatment even with a reduced (i.e., 50% lower) dose of each agonist and the upregulation of pro-inflammatory cytokine expression and recruitment and infiltration of the TME by immune cells. A remarkable finding of this study was that the level of CAFs in the TME was significantly reduced after immunotherapy with STING and TLR9 agonists.

CT-26 is a non-inflamed and cold tumor of the gastrointestinal tract exhibiting low levels of cytokine expression and a lack of immune cell infiltration (55). The underlying mechanism, according to our data, is that ADU-S100 combined with CpG ODN initiates local inflammation in the CT-26 tumor, which subsequently establishes systemic immune responses, for example, cellular immunity as observed in the TME and spleen. This result is consistent with previous findings that STING and TLR9 agonists promote robust antitumor inflammatory responses (8, 45–47). This revealed the promising outcomes of combination therapy in extending survival rate, inhibiting tumor growth, and suppressing tumorigenesis, accompanied by a stronger inflammatory state within the tumor and spleen due to the increased expression of pro-inflammatory cytokines, which play a key role during the initial inflammation and the transition to T cell-mediated immune responses.

In this study, for the first time, we present evidence of biomarkers of tumor progression inhibition in CT-26 colon carcinoma following immunoadjuvant therapy. To our knowledge, although several preclinical studies have investigated the efficiency and immunological mechanisms of innate immunoadjuvant therapy in preclinical colon carcinoma models (56–58), the effect of such treatment on CAFs’ infiltration of the TME has not been reported.

CAFs, as the key component of tumor stroma, play a significant role in many features of tumor biology, such as collagen deposition, tumor development, and immunosuppression (39).

CAFs are abundant in colorectal cancer and, within CT-26 tumors, constitute 25% of all live cells (55). CAFs, as important components of the TME, are associated with poor prognosis and therapeutic resistance (59).

Considering the upregulation of immune cell infiltration, cytokine production, and robust antitumor responses obtained by the combination of ADU-S100 and CpG ODN1826, we observed the downregulation of α-SMA and VIM as the main biomarkers of CAFs and predictive markers of cancer progression (**Figures 9**, **10**), suggesting that immunoadjuvant therapy employing STING and TLR9 agonists in single or combination forms suppresses the tumorigenesis potential of CAFs in the colon carcinoma model. Although there are alternative methods for detecting CAFs, α-SMA, as the most studied marker, is used for determining the heterogenicity and phenotype of CAFs in tumor types (35, 60). In this study, we observed that tumor growth inhibition is directly related to α-SMA and VIM expression. The combination of ADU-S100 and CpG ODN1826 exhibited the greatest downregulation of these markers, which was accompanied by the highest tumor inhibition rate (96%) in comparison to a single form of agonist (**Figure S2A** and **Figures 9** , **10**).

The significant decrease in the levels of CAFs in all treatment groups is probably due to the activation of the STING signaling pathway, as Kabashima et al. demonstrated that in pancreatic ductal adenocarcinoma tissues, the activation of the cyclic GMP-AMP synthase (cGAS)–STING pathway led to an increase in the markers of tumor-suppressing CAFs and the infiltration of tumor tissues by immune cells (34). The intravenous injection of a chemically modified STING agonist (diABZI) in an established CT-26 tumor model abolished 98% of the CD140b^+^ CAFs in the tumor stroma (55). Consistent with these data and our findings, the assumption relies on the interplay between the activation of the STING and TLR9 pathways and direct killing effects on CAFs in the TME. However, the underlying mechanisms of these findings have yet to be identified.

IL-6, as a cytokine produced by CAFs (39), demonstrated contrasting effects in our study. While monotherapy with ADU-S100 (20 µg) led to the downregulation of IL-6 compared with the control group, in the combination group the expression of IL-6 was significantly upregulated compared with the control group. This may be due to the direct effect of STING pathway activation on the inhibition of CAFs in the TME in monotherapy treatment, as in combination with CpG ODN1826, we found that IL-6 simultaneously diminished the abundance of CAFs and participated in the activation and proliferation of lymphocytes, as suggested in previous studies (6, 39, 61).

Type I and type II IFNs play a critical role in both innate and adaptive immunity and are essential for antitumor immune responses (62). The STING and TLR9 signaling pathways led to the production of type I IFNs in the TME (63, 64). Type I IFNs stimulate the apoptosis of tumor cells and prevent tumor cell proliferation and metastasis. The robust upregulation of IFN**-**β expression in the tumor and spleen tissue, along with the increased infiltration of CD8^+^ cells and reduced tumor cell mitosis observed in our study, was mainly due to the synergetic effect of STING and TLR9 agonists when administered in combination forms (**Figures 2A**, **3A**, **4F**, **8**). Type I IFNs enhance the accumulation of DCs in the TME and promote their maturation and migration into lymph nodes for activation of CD8^+^ T cells (65). In contrast to the spleen, in tumor tissues, the combined effects of STING and TLR9 agonists resulted in an approximately 70-fold increase in IFN**-**β expression compared with the control group, which is a remarkable finding and indicates the pro-inflammatory status in the TME (**Figures 2A**, **3A**).

The basal expression of type-I IFN by DCs resulted in the secretion of IFN-γ (6). Alternatively, activated T lymphocytes, as a result of the type I interferon signaling pathway, produce IFN-γ, which plays a crucial role in antitumor immunity (62). In our study, the tumoral expression of IFN**-γ** in the combination group was upregulated approximately twofold compared with the control group (**Figure 2B**). We also identified IL-12 as an important pro-inflammatory cytokine that regulates innate and adaptive immune responses and showed that IL-12 was upregulated in the tumor and spleen tissues of mice in all treatment groups (**Figures 3C**, **4C**) and that it may be effective in the induction of Th1 responses along with type I IFN production, in turn leading to the observed antitumor effects.

As a result of STING and TLR9 signaling pathways and the production of type I and II IFNs, the cross-priming of tumor antigens by CD8^+^ T cells facilitates antitumor immune responses (66–68). Our results suggest that the synergistic effects of immunoadjuvants toward polarization of T-cell-mediated immunity within the TME are evident by the IHC analysis of CD8^+^ cell infiltration and TNF-α expression (**Figures 8**, **2D**). As a hallmark cytokine in CD8^+^ T-cell activation and tumor infiltration (69), the synergistic effects of the combination treatment with both immunoadjuvants increased the tumoral expression of TNF-α twofold, whereas reduced expression of this cytokine was detected when single forms of immunoadjuvants were administered (**Figure 2D**). Consistent with this finding, the expression of CD8^+^ cells in the combination group was 59% higher than in the control group, which was remarkable compared with a single form of agonist (**Figure 8**). On the other hand, an increase in CDN concentration led to a decrease in T-cell infiltration of the tumor (70, 71). In support of these findings, our IHC results indicated that there was decreased infiltration by CD8^+^ cells in the ADU-S100 (40-µg) group. However, when a lower dose of ADU-S100 (20 µg) was used as a monotherapy or in the combination group, a greater than twofold increase in CD8^+^ cell infiltration of the TME was observed. These results are consistent with the expression of IFN-γ in the tumor. In addition, the expression of CD45, which has an essential function in regulating immune responses, showed similar level of upregulation in all groups treated with a single agonist. However, the synergy of the agonists led to a higher (twofold) increase in CD45 expression, which was probably due to the greater recruitment of T cells to the TME in this group (**Figure 7**). H&E staining, along with the expression of cleaved caspase-3 as a positive marker for the efficiency of cancer treatment (72), revealed that there was a remarkable increase in the apoptotic index of caspase-3 in the combination group, suggesting *in situ* cell apoptosis compared with treatment with single agonists (**Figure 6**).

Studies showed that STING and TLR9 agonists promote the activation of DCs in the spleen (6, 45). We showed that, despite the intratumoral injection of agonists, the upregulation of cytokines occurred in the spleen, indicating a systemic immune response to agonists. In the combination group, the splenic expression of all cytokines was significantly upregulated compared with the control (**Figure 3**). It probably indicates the additive effects of both agonists on the activation of immune cells within the secondary lymphoid tissues. However, the splenic expression of IFN-γ, IL-12, and TNF-α in the combination group showed different results. The expression of IFN-γ in the combination group was lower than in the ADU-S100 (40 µg) and CpG ODN (40-µg) groups. The CpG ODN (40 µg) group showed the highest level of splenic expression of IL-12, followed by the combination group. The splenic expression of TNF-α in the combination group was lower than in the ADU-S100 (20 µg) and ADU-S100 (40 µg) groups. These results might be due to the different inflammatory properties of immunoadjuvants in single or combination forms in secondary lymphoid tissues when they are administered locally in the tumor. Studies have shown that the intratumoral injection of the STING agonist developed systemic immune responses in the spleen and tumor-draining lymph nodes, which were indicated by increased levels of IFN-γ-producing CD8^+^T cells in these organs (73). In addition, in mice immunized with ovalbumin/STING/TLR7/8 agonists, the activation of antigen-specific CD8^+^ T cells in the spleen and lymph nodes was induced, which was significant compared with that observed in the control group, whereas a significant increase was not observed in the mice treated with a single form of agonist (74). Furthermore, this combination induces enhanced infiltration of T cells into distant tumors, indicating that systemic immune responses occurred (75). Contrary to these findings, we observed a lower level of splenic expression of IFN-γ, IL-12, and TNF-α in the combination group after local immunotherapy, and, therefore, this suggests that further studies are required to explore the detailed mechanisms behind the splenic expression of these cytokines after intratumoral immunotherapy with ADU-S100 and CpG ODN1826. In conclusion, our results demonstrated that monotherapy with either CpG ODN or ADU-S100 and a combination of both agonists induced tumor regression, extended survival, and reduced infiltration of the TME by CAFs, underscoring the potential of immunoadjuvant therapy in inhibiting CAF-induced tumorigenesis. Mechanistic studies can reveal how the STING and TLR9 signaling pathways modulate and inhibit CAFs’ tumorigenicity and immunosuppressive state. Therefore, CAF-mediated immune modulation may be considered a potential cellular target in immunotherapy strategies for colorectal cancer. Considering the heterogeneity and plasticity of CAFs and their crosstalk with immune and cancer cells, are key challenges that must be addressed for effective clinical translation of these findings.

## Data availability statement

The original contributions presented in the study are included in the article/**Supplementary Material**. Further inquiries can be directed to the corresponding author.

## Ethics statement

The animal study was approved under the ethical guidelines of the Animal Care and Use Committee of Ferdowsi University of Mashhad (license number 46013-M). The study was conducted in accordance with the local legislation and institutional requirements.

## Author contributions

SH: Data curation, Formal Analysis, Investigation, Methodology, Project administration, Software, Validation, Visualization, Writing – original draft, Writing – review & editing. SA: Data curation, Formal Analysis, Visualization, editing. ZM: Methodology. MG: Methodology. HF: Resources. AH: Conceptualization, Data curation, Formal Analysis, Funding acquisition, Investigation, Methodology, Project administration, Resources, Supervision, Validation, Writing – original draft, Writing – review & editing, Visualization, Software.

## Supplementary material

Supplementary Figure 1 The cell viability of CT-26 cells after 24 hours culture with different concentrations of agonists was determined by the MTT test (n=3). **(A)** ADU-S100, **(B)** CpG ODN, and **(C)** ADU-S100 (20μg/ml) + CpG ODN (20μg/ml).

Supplementary Figure 2
**(A)** Tumor inhibition rate in treated versus control groups, **(B)** comparison of spleen index (mg/g) among control and treatment groups, **(C)** Body weight changes in different treatment groups during the course of the experiment, (n=7). Data are represented as mean ±SD. Statistical significance differences were analyzed by Mann-Whitney tests and indicated as **p ≤ 0.01 and *p ≤ 0.05).

Supplementary Figure 3
**(A)** Hemorrhage (thin arrows) and necrosis (star) in the control group. Necrotic cells have pyknotic (green arrows) and karyorrhectic (yellow arrows) nuclei or have lost their nucleus with highly eosinophilic cytoplasm. **(B–E)** Tumor tissues in the treatment groups without prominent hemorrhage and necrosis representing tumor malignancy. **(B)** ADU-S100 (20µg), **(C)** ADU-S100 (40µg), **(D)** CpG ODN (40µg), and **(D)** ADU-S100 (20µg) + CpG ODN (20µg). Scale bars = 50μm. (Hematoxylin-eosin staining, ×400 magnification).

Supplementary Figure 4 Increased number of lymphocytes (%) in blood samples obtained in control and treatment groups on day 30th (n=7). Data are represented as mean ±SD. Statistical significance differences were analyzed by Mann-Whitney tests and indicated as **p ≤ 0.01.
