# Supplementary figures and images for: Immunotherapy with STING and TLR9 agonists promotes synergistic therapeutic efficacy with suppressed cancer-associated fibroblasts in colon carcinoma
Source: Front Immunol. 2023 Oct 13;14:1258691. doi: 10.3389/fimmu.2023.1258691 (PMC10611477; doi:10.3389/fimmu.2023.1258691)

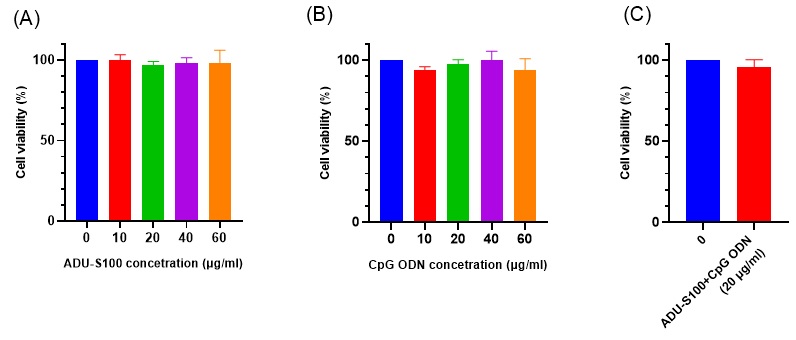

Supplement: Supplementary Figure 1 — The cell viability of CT-26 cells after 24 hours culture with different concentrations of agonists was determined by the MTT test (n=3). (A) ADU-S100, (B) CpG ODN, and (C) ADU-S100 (20μg/ml) + CpG ODN (20μg/ml). [file Image_1.jpg]

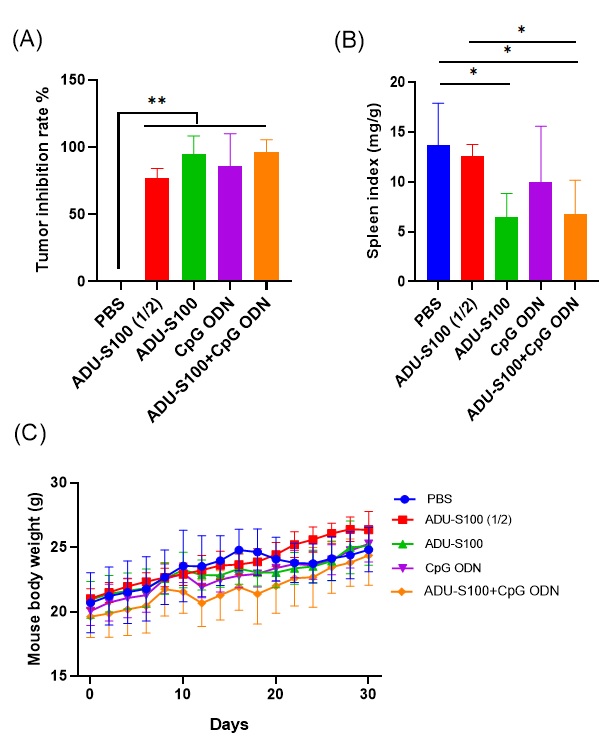

Supplement: Supplementary Figure 2 — (A) Tumor inhibition rate in treated versus control groups, (B) comparison of spleen index (mg/g) among control and treatment groups, (C) Body weight changes in different treatment groups during the course of the experiment, (n=7). Data are represented as mean ±SD. Statistical significance differences were analyzed by Mann-Whitney tests and indicated as **p ≤ 0.01 and *p ≤ 0.05). [file Image_2.jpg]

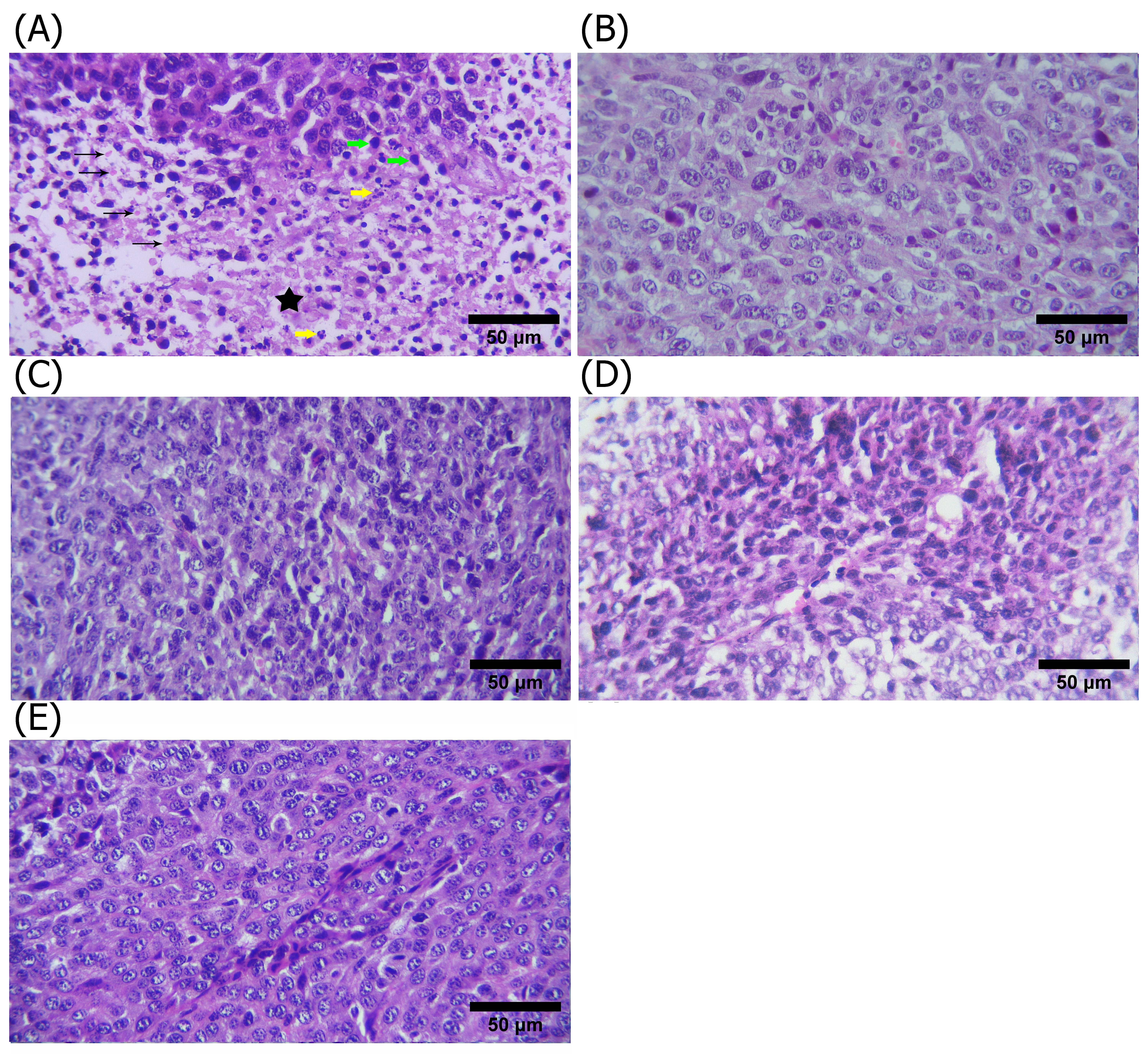

Supplement: Supplementary Figure 3 — (A) Hemorrhage (thin arrows) and necrosis (star) in the control group. Necrotic cells have pyknotic (green arrows) and karyorrhectic (yellow arrows) nuclei or have lost their nucleus with highly eosinophilic cytoplasm. (B–E) Tumor tissues in the treatment groups without prominent hemorrhage and necrosis representing tumor malignancy. (B) ADU-S100 (20µg), (C) ADU-S100 (40µg), (D) CpG ODN (40µg), and (D) ADU-S100 (20µg) + CpG ODN (20µg). Scale bars = 50μm. (Hematoxylin-eosin staining, ×400 magnification). [file Image_3.jpg]

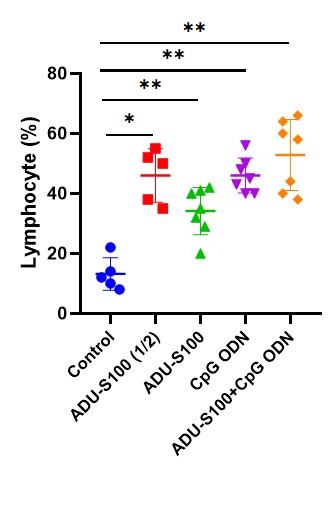

Supplement: Supplementary Figure 4 — Increased number of lymphocytes (%) in blood samples obtained in control and treatment groups on day 30th (n=7). Data are represented as mean ±SD. Statistical significance differences were analyzed by Mann-Whitney tests and indicated as **p ≤ 0.01. [file Image_4.jpg]
